# MicroRNA-124 regulates the expression of MEKK3 in the inflammatory pathogenesis of Parkinson’s disease

**DOI:** 10.1186/s12974-018-1053-4

**Published:** 2018-01-12

**Authors:** Longping Yao, Yongyi Ye, Hengxu Mao, Fengfei Lu, Xiaozheng He, Guohui Lu, Shizhong Zhang

**Affiliations:** 1 Department of Neurosurgery, Zhujiang Hospital, Southern Medical University, Guangzhou, 510282 China; 2 The National Key Clinic Specialty, The Engineering Technology Research Center of Education Ministry of China, The Neurosurgery Institute of Guangdong Province, Guangdong Provincial Key Laboratory on Brain Function Repair and Regeneration, Southern Medical University, Guangzhou, 510282 China; 3 Department of Neurosurgery, The First Affiliated Hospital of Nanchang University, Nanchang, 330006 China

**Keywords:** Parkinson’s disease, MicroRNA-124, Microglia, MEKK3, NF-κB

## Abstract

**Background:**

Parkinson’s disease (PD) is the most prevalent neurodegenerative disorder that is characterised by selective loss of midbrain dopaminergic (DA) neurons. Chronic inflammation of the central nervous system is mediated by microglial cells and plays a critical role in the pathological progression of PD. Brain-specific microRNA-124 (miR-124) expression is significantly downregulated in lipopolysaccharide (LPS)-treated BV2 cells and in the 1-methyl-4-phenyl-1,2,3,6-tetrahydropyridine (MPTP) model of PD. However, whether abnormal miR-124 expression could regulate the activation of microglia remains poorly understood.

**Methods:**

BV2 cells were activated by exposure to LPS, and the expression levels of miR-124, mitogen-activated protein kinase kinase kinase 3 (MEKK3), and the nuclear factor of kappaB (NF-κB) p-p65 were analysed. Over-expression and knockdown studies of miR-124 were performed to observe the effects on MEKK3/NF-κB signalling pathways, and the induction of pro-inflammatory and neurotoxic factors was assessed. In addition, a luciferase reporter assay was conducted to confirm whether MEKK3 is a direct target of miR-124. Meanwhile, production of miR-124, MEKK3, and p-p65; midbrain DA neuronal death; or activation of microglia were analysed when treated with or without miR-124 in the MPTP-induced model of PD.

**Results:**

We found that the knockdown of MEKK3 could inhibit the activation of microglia by regulating NF-κB expression. Over-expression of miR-124 could effectively attenuate the LPS-induced expression of pro-inflammatory cytokines and promote the secretion of neuroprotective factors. We also first identified a unique role of miR-124 in mediating the microglial inflammatory response by targeting MEKK3/NF-κB signalling pathways. In the microglial culture supernatant (MCS) transfer model, over-expression of the miR-124 or knockdown of MEKK3 in BV2 cells prevented SH-SY5Y from apoptosis and death. Moreover, MEKK3 and p-p65 were abundantly expressed in the midbrain. Furthermore, their expression levels increased and microglial activation was observed in the MPTP-induced model of PD. In addition, exogenous delivery of miR-124 could suppress MEKK3 and p-p65 expression and attenuate the activation of microglia in the substantia nigra pars compacta of MPTP-treated mice. miR-124 also could prevent MPTP-dependent apoptotic midbrain DA cell death in a MPTP-induced PD model.

**Conclusions:**

Taken together, our data suggest that miR-124 can inhibit neuroinflammation in the development of PD by regulating the MEKK3/NF-κB signalling pathways and implicate miR-124 as a potential therapeutic target for regulating the inflammatory response in PD.

## Background

Parkinson’s disease (PD) is one of the most common neurodegenerative diseases worldwide, and its clinical features are characterised by the progressive degeneration of midbrain dopaminergic (DA) neurons in the substantia nigra pars compacta (SNpc) [1]. Currently, no effective specialised treatment has been developed for this pathology, and the cause of the neurodegeneration is still unknown. Chronic inflammation in the central nervous system (CNS) plays a critical role in the pathological progression of PD. Microglia, a type of neuroglia, are macrophages in the CNS and are the chief resident immune cells in the brain, where they act as the main active immune defence [2]. The presence of activated microglial cells within the substantia nigra has been reported in post-mortem studies and in a 1-methyl-4-phenyl-1,2,3,6-tetrahydropyridine (MPTP)-induced animal model of PD [3]. Impaired or dead midbrain DA neurons can directly induce the activation of microglia, increasing the production of ROS and pro-inflammatory cytokines [4]. The activated microglia will produce many inflammatory cytokines that contribute to midbrain DA cell apoptosis and death [5]. Control of microglial activation might help to increase neuronal survival and mitigate PD [6].

MicroRNAs (miRNAs) are small non-coding RNA molecules that play a complex role in the regulation of transcription of multiple genes via binding to 3′ untranslated regions (3′-UTR) [7]. Emerging evidence demonstrates that post-transcriptional regulation by miRNA machinery plays an important role in PD pathogenesis [8, 9]. For example, miR-433 inhibits the translation of FGF20, which has previously been shown to cause PD through both over-expression and point mutations [10]. In addition, miR-7 protects against MPTP-induced cell death by promoting glycolysis and targeting RelA (p65) [11, 12]. Specifically, sustained aberrant miRNA expression levels have been described in inflammatory and immune-related neurodegenerative disorders [13]. Several different microRNAs have been implicated as important regulators and fine-tuners of immune system activation and neuroinflammation. miR-155 is required for α-syn-induced inducible nitric oxide synthase (iNOS) expression in microglia in models of PD [14], while three of these miRNAs (miR-125b-5p, miR-342-3p, and miR-99a) were specifically expressed in microglia [15].

MicroRNA-124 (miR-124) is highly expressed in the brain, with an abundance that is (more than 100 times) higher than that in other organs, and it plays a critical role in PD, which regulates apoptosis and autophagy in the MPTP model of PD by targeting Bim and loads nanoparticles to enhance brain repair in PD [16–18]. In fact, miR-124 promotes microglial quiescence, and knockdown of miR-124 in microglia resulted in its activation [19]. Furthermore, miR-124 mediates cholinergic anti-inflammatory action by inhibiting the production of pro-inflammatory cytokines [20]. However, whether miR-124 could attenuate microglial activation in the development of PD remains unknown.

MEKK3, a member of the mitogen-activated protein kinase kinase kinase (MAP3K), has also been suggested to play a major role in the inflammation response, including in the process of inducing nuclear factor of kappaB (NF-κB) activation [21, 22]. To date, only few studies have been conducted about the role of MEKK3 in PD. For example, recent research has shown that HtrA serine peptidase 2 (HtrA2) is phosphorylated upon MEKK3 activation in PD [23]. In addition, increased colocalisation of NF-κB has been demonstrated in the SNpc of post-mortem PD brains [24]. Activation of NF-κB plays an important role in the loss of midbrain DA cells in MPTP-intoxicated mice and PD patients [25, 26]. Even so, little is known about the role of MEKK3 in inflammatory pathogenesis in PD and it remains unclear whether MEKK3 could mediate NF-κB activation in microglia. Hence, in our study, we provide a direct correlation between miR-124 and MEKK3/NF-κB signalling pathways in the inflammatory pathogenesis of PD in vivo and in vitro.

## Methods

### Animals and treatment

Ten-week-old male C57BL/6 mice were purchased from the Sun Yat-sen University Laboratory Animal Center. The animals were accommodated in a controlled environment and supplied with standard rodent chow and water, and investigators were blinded to the experimental treatment. The mice received one intraperitoneal injection of MPTP-HCl per day (30 mg/kg free base; CAS23007-85-4; Sigma, MO, USA) for five consecutive days, while control mice received saline injections. The mice were decapitated, and once the brain was removed, the ventral midbrain, which contained the SNpc, was dissected and stored at − 80 °C for further study. Regarding the experiment with the exogenous delivery of miR-124 into an animal model, the right lateral ventricle of the mice was surgically implanted with a stereotactic catheter (62004, 62104, and 62204; Woruide, Shenzhen, China). Before the stereotactic intraventricular injection, mice were intraperitoneally anaesthetised by pentobarbital sodium (60 mg/kg). The stereotactic intraventricular injection site was chosen as previously reported (anterior-posterior − 0.5 mm, medial-lateral − 0.7 mm, dorsoventral − 2.9 mm) [27]. After the injections, the mice were kept warm (37 °C) until they recovered from surgery (1 week). The mice were then administered one dose of agomir (MIMAT0000134; RiboBio, Guangzhou, China) miR-124-3p (20 nM of ribonucleotide in a total volume of 5 μL) through the catheter per day for five consecutive days. Agomir-negative control sequences (MIMAT0000039) were injected into the right lateral ventricle as the negative control. The agomir treatment was performed 2 days prior to the injection of MPTP.

### Cell culture and treatment

BV2 microglial cells and SH-SY5Y cells were obtained from the Central Laboratory of Nanfang Hospital (Guangzhou, China). BV2 cells were maintained in DMEM (Gibco, Carlsbad, CA, USA), supplemented with 10% heat-inactivated foetal bovine serum (Gibco) and 0.1% penicillin-streptomycin (Sigma-Aldrich, St. Louis, MI, USA). Human midbrain DA cell line SH-SY5Y cells were maintained in DMEM supplemented with 10% FBS (Gibco) and 0.1% penicillin-streptomycin. Both cells were cultured at 37 °C in a humidified incubator with 95% air/5% CO_2_. The reagents for cell culture in our research have been reported in previous studies [28, 29].

### Transfection

The miR-124 mimics (miR10000134), control miRNA mimics (MIMAT0000295), miR-124 inhibitor (miR20000134), and control inhibitors (MIMAT0000039) were synthesised by RiboBio (Guangzhou, China) and were transfected into BV2 cells by using riboFECT™ CP (RiboBio) according to the manufacturer’s protocol. The miRNA-124 mimic sequence was 5′-CCGUAAGUGGCGCACGGAAU-3′. The miR-124 inhibitor sequence was 5′-GGCAUUCACCGCGUGCCUUA-3′. In addition, the small interfering RNA (siRNA) specifically targeting MEKK3 (MEKK3 siRNA) and the control siRNA (siNC) were purchased from Shanghai GenePharma (A10001; Shanghai, China). MEKK3 siRNA and siNC were transfected with Lipofectamine 2000 Reagent (11668019; Invitrogen, USA) according to the manufacturer’s instructions. The sense strand of the MEKK3 siRNA was 5′-GGAGAGACGAAUUAUAGCATT-3′, and the antisense strand was 5′-UGCUAUAAUUCGUCUCUCCTT-3′. The recommended concentrations are as follows: miR-124 mimics (50 nM), miR-124 inhibitor (100 nM), and MEKK3 siRNA (100 nM).

### Reverse transcription quantitative real-time polymerase chain reaction

Total RNA was extracted with TRIzol reagent (15596018; Invitrogen, Carlsbad, CA, USA) according to the manufacturer’s instructions. For the messenger RNA (mRNA) quantification of the protein-encoding genes, RNA was reverse transcribed to complementary DNA (cDNA) with a random primer (Sangon Biotech, Shanghai, China) using a Reverse Transcription Kit (RR047A; Takara, Dalian, China), and the mRNA levels were determined using reverse transcription quantitative real-time polymerase chain reaction (RT-qPCR). A primer pair for the detection of mouse glyceraldehyde-3-phosphate dehydrogenase (GAPDH) was used as the internal control. RT-qPCR for the detection of miR-124 was performed using miR-124-specific PCR primers (RiboBio) with PrimeScript RT Master Mix (5×) and SYBR Premix Ex Taq™ II (RR047A and RR820A; Takara, Dalian, China) according to the manufacturer’s instructions, normalised to U6 snRNA. The relative expression of each gene was calculated and normalised using the ΔΔCt method. All the sequences of the primers used are as follows:

**Table:** 

Genes	Primer sequences
miR-124	5′-GCGAGGATCTGTGAATGCCAAA-3′
U6	5′-GCTTCGGCAGCACATATACTAAAAT-3′
GAPDH	Forward: 5′-GGGAAATTCAACGGCACAGT-3′
	Reverse: 5′-AGATGGTGATGGGCTTCCC-3′
MEKK3	Forward: 5′-TGTACCTGAGCGACAACAGC-3′
	Reverse: 5′-CACTGCTGAGGGGATCTAGC-3′
TNF-α	Forward: 5′-TATGGCTCAGGGTCCAACTC-3′
	Reverse: 5′-GGAAAGCCCATTTGAGTCCT-3′
IL-6	Forward: 5′-TTCCATCCAGTTGCCTTCTT-3′
	Reverse: 5′-CATTTCCACGATTTCCCAGA-3′
iNOS	Forward: 5′-GCTTGGGTCTTGTTCACTCC-3′
	Reverse: 5′-TCCTCTTTCAGGTCACTTTGG-3′
TGF-β1	Forward: 5′-GCACGTGGAGCTGTACCA-3′
	Reverse: 5′-CAGCCGGTTGCTGAGGTA-3′
IL-10	Forward: 5′-GCCTTATCGGAAATGATCCA-3′
	Reverse: 5′-AGGGTCTTCAGCTTCTCACC-3′

### Western blot analysis

A western blotting assay was performed to detect the protein level of MEKK3 and p-p65 in cultured cells and selected mouse midbrains. Total protein was extracted using radioimmunoprecipitation assay (RIPA) lysis buffer (P0013B; Beyotime, Jiangsu, China) with protease and phosphatase inhibitors (B14001 and B15001; BioTools, Olathe, KS, USA), following the manufacturer’s protocol. The protein concentration was measured with bicinchoninic acid (BCA) protein assay (Bio-Rad Laboratories, Inc., Berkeley, CA, USA). Equal amounts of protein were isolated using sodium dodecyl sulphate polyacrylamide gel electrophoresis (Beyotime Biotechnology, Shanghai, China) and then transferred to a polyvinylidene fluoride membrane (IPVH00010; Millipore, Bedford, USA). After blocking in 5% Tris-buffered saline-Tween, the membrane was incubated with primary antibody at 4 °C overnight. The antibodies used were as follows: rabbit anti-MEKK3 (NB100-92399; Novus, USA), mouse anti-p-p65 (Ser536; Cell Signaling Technology, USA), rabbit anti-GAPDH (ab8245; Abcam, Cambridge, MA, USA), goat anti-rabbit IgG-HRP (31460; Life Technologies, USA), and rabbit anti-mouse IgG (A-21065; Life Technologies).

### Immunohistochemical analysis and quantitative evaluation

Mice were deeply anaesthetised with a pentobarbital and transcardially perfused with saline followed by 4% polyformaldehyde-HCl, and the midbrains were selected. Immunostaining was performed as previously described [30]. The total number of Iba1-positive cells, tyrosine hydroxylase (TH)-positive neurons, and apoptotic neurons in the SNpc of selected mice was counted as previously described [31]. The MEKK3 and Iba1^+^ integrated optical density (IOD) were determined by using Image-Pro Plus software (Media Cybernetics, Silver Spring, USA). The antibodies used were as follows: rabbit-derived anti-Iba1 (019-19741; 1:500; Wako Chemicals, Japan), rabbit anti-MEKK3 (NB100-92399; 1:100; Novus, USA), rabbit anti-TH (GB11181; 1:100; Servicebio, Wuhan, China), and HRP-labelled goat anti-rabbit IgG (GB23303; 1:500; Servicebio).

### Double immunofluorescence staining and confocal laser scanning microscopy

The procedures for double immunofluorescence staining were performed as that in our previous report [32]. In brief, free-floating 30-μm sections of the midbrain from each group were incubated with rabbit-derived anti-Iba1 (019-19741; 1:500; Wako Chemicals), rabbit anti-MEKK3 (NB100-92399; 1:100; Novus), and rabbit anti-TH (GB11181; 1:100; Servicebio) at 4 °C overnight followed by Cy3-conjugated goat anti-rabbit IgG (GB21303, 1:300; Servicebio) or a goat anti-mouse IgG conjugated with Alexa Fluor 488 (GB25301, 1:400; Servicebio) for 1 h at room temperature (22 ± 2 °C). Viewed under a LSM 880 (Carl Zeiss, Jena, Germany) laser scanning confocal microscopy, immunoreactivity exhibited green or red fluorescence. Confocal images were acquired and analysed using ZEN lite software (Carl Zeiss).

### Luciferase reporter assay

According to the TargetScan database, miR-124 potentially binds to MEKK3. To construct luciferase reporter vectors, the 3′-UTR of MEKK3 cDNA fragments containing the predicted potential miR-124 binding sites were subcloned into the XhoI/NotI site of psi-CHECK™-2 Vector (Promega, Madison, WI, USA). The constructs were cotransfected into HEK293 cells along with scramble (50 nM) or miR-124 mimic (50 nM) using riboFECT™ CP as described by the manufacturer. Luciferase activities were measured with the Dual-Luciferase Reporter Assay System (Promega) 48 h after transfection. Renilla luciferase activity was normalised to that of firefly luciferase.

To observe the NF-κB activity, luciferase assay was performed as previously described [33]. The cells in 12-well plates were cotransfected with 0.5 μg NF-κB-responsive luciferase reporter plasmid containing four κB sites (pNF-κB-Luc; Clontech) and 0.2 μg pSV-β-galactosidase expression plasmid (Promega). After 24 h, cells were treated with different experimental conditions, and luciferase activities were analysed using a luminometer and then normalised with β-galactosidase activity.

### Microglial culture supernatant transfer model

To test the neurotoxic effects of activated microglia, confluent SH-SY5Y cells were cultured in complete medium and treated with the supernatants of lipopolysaccharide (LPS)-stimulated BV2 cells. To study the effects of different transfection materials, the BV2 cells were exposed in the presence or absence of various materials for 48 h and then stimulated with the 1 μg/mL LPS for another 12 h. The resulting culture supernatants were collected, centrifuged to eliminate cell debris, and transmitted to SH-SY5Y cells for 12 h to induce cell apoptosis and death. The cells were then harvested and washed three times with PBS buffer.

### Flow cytometry analysis

The apoptosis of the SH-SY5Y cells that were harvested in the microglial culture supernatant (MCS) transfer model was quantified using an annexin V-fluorescein isothiocyanate/propidium iodide (PI) apoptosis detection kit (Dojindo, Tokyo, Japan). The cells were pelleted and resuspended in 5 μL fluorescein isothiocyanate-labelled annexin V (V-FITC), and 5 μL propidium iodide staining solution was added to the cells, followed by incubation at room temperature (shielded from light) for 10 min. The number of apoptotic cells was assayed with flow cytometry (FACSVerse; Becton Dickinson, CA, CT, USA).

### Statistical analysis

All data were presented as the mean ± SD from three independent experiments. Statistical analysis was carried out using one-way ANOVA and two-tailed Student’s *t* test. *P* < 0.05 was considered to indicate a statistically significant difference.

## Results

### Brain-specific miR-124 was downregulated in the LPS-stimulated BV2 cells

**Fig. 1:**
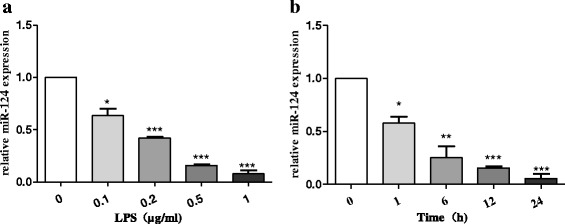
Brain-specific miR-124 was downregulated in the LPS-stimulated BV2 cells. miR-124 expression level was determined using reverse transcription quantitative real-time PCR (RT-qPCR) and normalised with U6 RNA. **a** miR-124 expression in BV2 cells treated with different concentrations (0.1, 0.2, 0.5, and 1 μg/mL) of LPS for 24 h. **b** miR-124 expression in BV2 cells exposed to 1 μg/mL LPS for different durations (0, 1, 6, 12, and 24 h). The data are shown as the mean ± SE from three independent experiments. The fold change is statistically significant. The fold change is significant where *P < 0.05, **P < 0.01, and ***P < 0.001

Considering that miR-124 is highly expressed in the brain and promotes microglial quiescence [17, 19], we investigated the expression of miR-124 in the cell lines by RT-qPCR. To compare miR-124 levels in both resting and active microglia, BV2 microglial cells were incubated with LPS. With the stimulation of an increasing concentration gradient of LPS (0.1, 0.2, 0.5, and 1 μg/mL) for 24 h, we found that the miR-124 expression showed a significant dose-dependent induction (Fig. 1a). Following the stimulation of BV2 cells with LPS (1 μg/mL) maintaining a temporal gradient (0, 1, 6, 12, and 24 h), the downtrend in the miR-124 level was monitored by RT-qPCR at different time points, and this effect became much more pronounced at 24 h (Fig. 1b). All these results showed a significantly lower level of miR-124 in LPS-stimulated BV2 than in the controls.

### Over-expression of miR-124 could effectively attenuate LPS-induced BV2 microglial activation

**Fig. 2:**
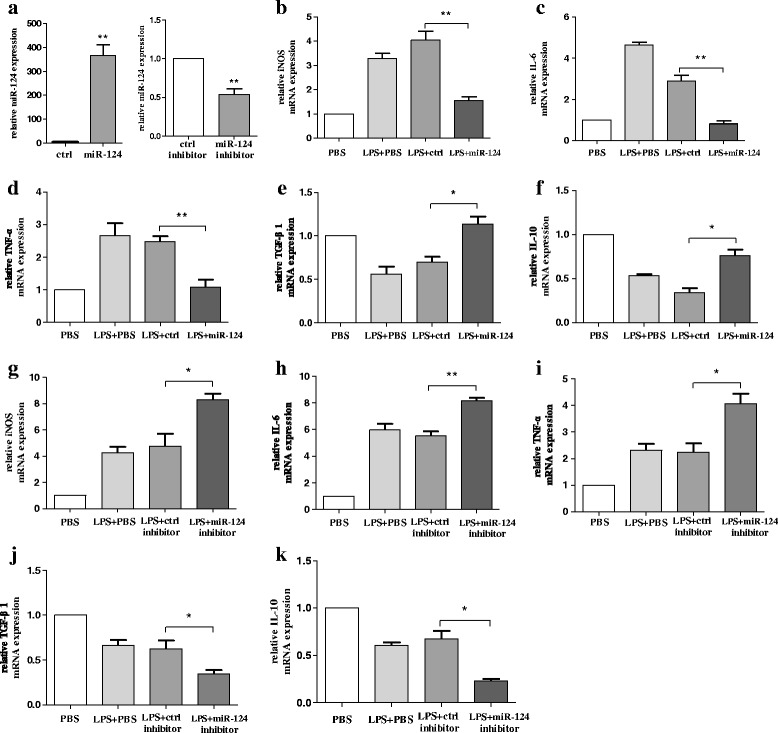
Over-expression of miR-124 could effectively attenuate LPS-induced BV2 microglial activation. **a** Microglia were transfected with miR-124 mimics or miR-124 inhibitor. After 48 h, cells were harvested, and miR-124 expression was evaluated using RT-qPCR. **b**–**f** BV2 cells were transfected with miR-124 mimics or miR-124 control for 48 h and then treated with LPS. After 24 h, cells were harvested, and the mRNA levels of the pro-inflammatory cytokines iNOS (**b**), IL-6 (**c**), TNF-α (**d**), TGF-β1 (**e**), and IL-10 (**f**) were evaluated using RT-qPCR. **g**–**k** BV2 cells were transfected with miR-124 inhibitor or control inhibitor for 48 h. The mRNA levels of pro-inflammatory cytokines iNOS (**g**), IL-6 (**h**), TNF-α (**i**), TGF-β1 (**j**), and IL-10 (**k**) were determined using RT-qPCR following a 24-h incubation with LPS. The fold change was normalised by GAPDH RNA levels. Experiments performed in triplicate showed consistent results. Data are presented as the mean ± SD. The fold change is statistically significant. *P < 0.05, **P < 0.01

We first demonstrated that the expression level of miR-124 decreased in the activated BV2 cells. The downregulation of miR-124 in response to LPS stimulation suggested that miR-124 might be involved in the regulation of the microglial response to LPS. We then determined whether the over-expression of miR-124 could attenuate LPS-induced BV2 microglial activation. To assess this issue, the over-expression of miR-124 in microglia was examined, and the transfection efficacy of miR-124 mimics and miR-124 inhibitor was assessed using RT-qPCR (Fig. 2a). However, over-activated microglia can mediate the detrimental effects of neurotoxicity and inflammation through the excess production of cytotoxic factors such as iNOS, TNF-α, and IL-6 [34, 35]. Moreover, TGF-β1 and IL-10 have been reported to mediate the inhibitory effects on microglial activation, often referred to as anti-inflammatory cytokines [36]. We then investigated the effects of modulated miR-124 expression in microglia transfected with miR-124 mimic and miR-124 inhibitor. TNF-α, iNOS, IL-6, TGF-β1, and IL-10 mRNA levels were determined using RT-qPCR following a 24-h incubation with LPS. As a result, we showed that the neurotoxic cytokines iNOS, IL-6, and TNF-α had strongly decreased mRNA levels following the over-expression of miR-124 (Fig. 2b–d), whereas TGF-β1 and IL-10, for which the production was decreased in the LPS-induced BV2 cells, had significantly increased mRNA levels with the over-expression of miR-124 (Fig. 2e, f). In addition, the mRNA expression levels of the pro-inflammatory cytokines iNOS, IL-6, and TNF-α were highly increased while the neuroprotective factors TGF-β1 and IL-10 decreased meaningfully when transfected with the miR-124 inhibitor (Fig. 2g–k). Taken together, the over-expression of miR-124 could effectively suppress LPS-induced microglial activation.

### Knockdown of MEKK3 suppresses the expression of p-p65 and the secretion of pro-inflammatory cytokines in BV2 cells

**Fig. 3:**
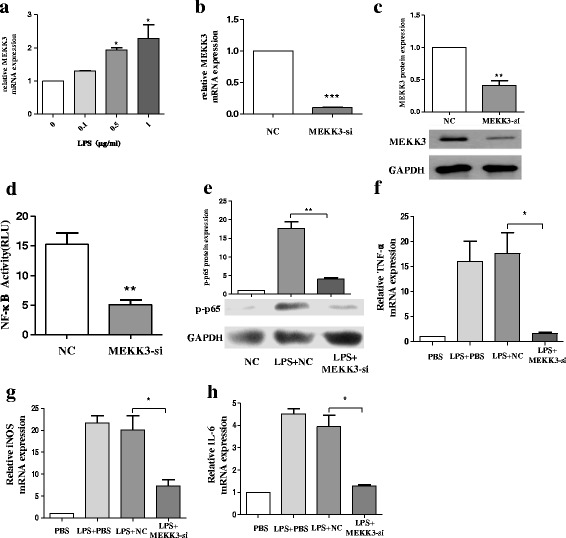
Knockdown of MEKK3 suppresses the expression of p-p65 and the secretion of pro-inflammatory cytokines in BV2 cells. **a** BV2 cells were treated with an increasing concentration of LPS (0.1, 0.5, and 1 μg/mL). After 12 h, cells were harvested for RNA isolation. Then, RT-qPCR analysis detected changes in the transcript level of MEKK3. **b**, **c** BV2 cells were transfected with negative control (NC) or MEKK3 siRNA (MEKK3-si). After 48 h, cells were harvested, and the MEKK3 mRNA (**b**) and protein expression (**c**) levels were evaluated using RT-qPCR and western blot analysis. **d**, **e** BV2 cells were transfected with NC or MEKK3-si for 48 h. Cells were washed with PBS and then stimulated with LPS (1 μg/mL) for 12 h. Cells were harvested. NF-κB activity was analysed by luciferase assay (**d**). Western blotting confirmed the protein expression of p-p65 (**e**). **f**–**h** BV2 cells were transfected with NC or MEKK3-si. After 48 h, cells were washed with PBS and then treated with LPS (1 μg/mL), After 12 h, the cells were harvested. The mRNA levels of pro-inflammatory cytokines TNF-α (**f**), iNOS (**g**), and IL-6 (**h**) were examined by RT-qPCR. GAPDH was used as a loading control for normalising the image density. The data are shown as the mean ± SE from three independent experiments. The fold change is statistically significant where *P < 0.05, **P < 0.01, and ***P < 0.001

MEKK3 is a member of the MAP3K superfamily and can mediate NF-κB activation [21, 22]. MEKK3 is constitutively expressed in both innate and adaptive immune cells such as T cells [37] and macrophages [38], but little is known about the role of MEKK3 in microglial activation. We first investigated the expression of MEKK3 in both resting and active microglia by RT-qPCR. The treatment of BV2 cells with increasing concentrations of LPS (0.1, 0.5, and 1 μg/mL) for 12 h showed significantly increasing expression of MEKK3 compared with the control group (Fig. 3a). In the next series of experiments, we asked whether MEKK3 could regulate microglial activation. We transfected MEKK3 siRNA (MEKK3-si), and the efficiency of the knockdown was evaluated by RT-qPCR and western blotting in BV2 cells (Fig. 3b, c). The MEKK3 knockdown constructs resulted in lower activity of NF-κB as analysed by luciferase assay compared with the negative control (NC) group (Fig. 3d). Western blotting showed decreased expression in protein levels of phosphorylation of NF-κB p65 (p-p65) in the MEKK3-si group (Fig. 3e). To investigate the precise effect of MEKK3 on LPS-induced inflammatory cytokine secretion in vitro, we impaired the expression of MEKK3 in BV2 cells, using siRNA specific for MEKK3. BV2 cells were transfected with the MEKK3 siRNA or NC for 48 h and were then stimulated with LPS (1 μg/mL) for 12 h. After treatment, the TNF-α, iNOS, and IL-6 expression levels were examined by RT-qPCR. Compared with the NC group, the result showed that the expression levels of TNF-α, iNOS, and IL-6 were significantly lower in MEKK3 knockdown BV2 cells (Fig. 3f– h).

### miR-124 targets MEKK3

**Fig. 4:**
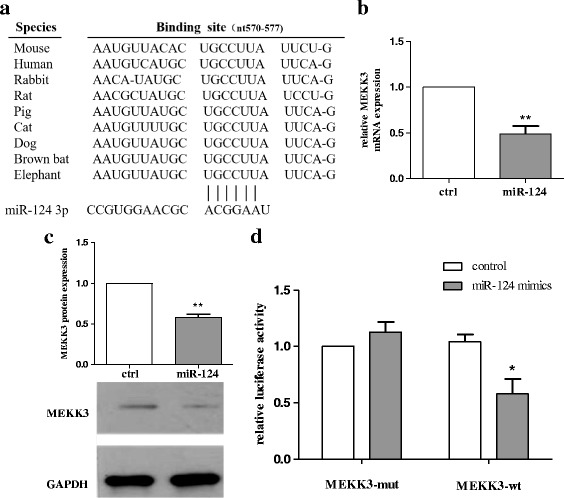
miR-124 targets MEKK3. **a** Alignment of the miR-124 binding site to Bim 3′-UTR is shown for different species as predicted using the TargetScan database. **b**, **c** BV2 cells were transfected with miR-124 mimics or ctrl mimics. After 48 h, cells were harvested, and the expression levels of MEKK3 mRNA (**b**) and protein (**c**) were evaluated using RT-qPCR and western blot analysis. The fold change was normalised by GAPDH levels. **d** Luciferase activity in HEK293 cells transfected with reporter constructs containing wild-type (WT) or mutated Bim 3′-UTR. The cells were cotransfected with indicated constructs and miR-124 mimics (100 nm) or control, and normalised levels of luciferase activity are shown. The results are presented as the mean ± SE from three independent experiments. The fold change is statistically significant. *P < 0.05, **P < 0.01. NS not significant

In general, miRNAs can target the 3′-UTR of target mRNAs as the post-transcription regulators of gene expression [7]. Having found that miR-124 inhibited inflammation in the BV2 cells, we investigated the mechanisms underlying this effect. We used the TargetScan database (Whitehead Institute, Cambridge, MA, USA) to analyse the targets predicted for miR-124. Computational prediction via the TargetScan database revealed that highly and poorly conserved miR-124 (miR-124-3p) might target the 3′-UTR of MEKK3 (Fig. 4a). When we transfected miR-124 mimics to BV2 cells, compared with the control, MEKK3 expression was reduced in both its mRNA (Fig. 4b) and protein levels (Fig. 4c). Then, we detected that miR-124 directly binds to the mRNA encoding MEKK3 by using the luciferase reporter system. Indeed, the luciferase activity reduced significantly when cells were transfected with miR-124 mimics (Fig. 4d).

### miR-124 attenuates LPS-induced inflammatory responses by targeting MEKK3/NF-κB signalling pathways in BV2 cells

**Fig. 5:**
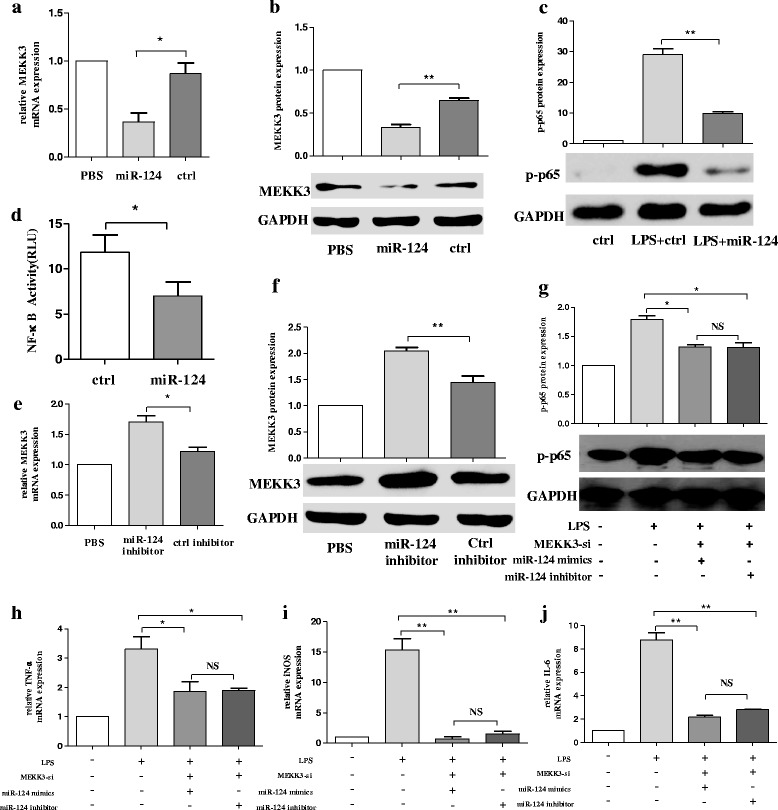
miR-124 attenuates LPS-induced inflammatory responses by targeting MEKK3/NF-κB signalling pathways in BV2 cells. The BV2 cells were transfected with miR-124 mimics or ctrl mimics. After 48 h, the production mRNA levels of MEKK3 (**a**) were determined using RT-qPCR, and western blot analysis was used to analyse the protein expression changes of MEKK3 (**b**) and p-p65 (**c**). NF-κB activity was analysed using luciferase assay (**d**). **e**, **f** The BV2 cells were transfected with miR-124 inhibitor or ctrl inhibitor, and the cells were harvested at 48 h. The relative expression of MEKK3 mRNA was evaluated using RT-qPCR (**e**), and the MEKK3 protein expression was studied using western blot analysis (**f**). **g**–**j** The BV2 cells were pre-transfected with MEKK3 siRNA for 48 h, and then the cells were transfected with miR-124 mimic or miR-124 inhibitor. After 24 h, cells were harvested, and the expression levels of p-p65 protein were evaluated using western blot analysis (**g**). RT-qPCR confirmed the expression of pro-inflammatory cytokines of TNF-α (**h**), iNOS (i), and IL-6 (**j**). The data were normalised against GAPDH. Data are normalised to saline controls and presented as the mean ± SD of three independent experiments. The fold change is statistically significant. *P < 0.05, **P < 0.01. NS not significant

As stated earlier, we found that the knockdown of MEKK3 suppressed the activation of NF-κB and the secretion of pro-inflammatory cytokines in BV2 cells. We further studied whether miR-124 could attenuate LPS-induced expression of MEKK3. Following the transfection of miR-124 mimics in BV2 cells, the p-p65 protein level and MEKK3 mRNA and protein levels were both significantly inhibited as compared with the control (Fig. 5a–c). Meanwhile, luciferase assay showed a significant decrease in NF-κB activity (Fig. 5d). By contrast, the production of MEKK3 increased prominently in terms of both mRNA and protein levels when the BV2 cells were transfected with miR-124 inhibitor (Fig. 5e, f). To investigate whether the inhibitory effect on NF-κB of miR-124 was through MEKK3, the BV2 cells were transfected with MEKK3 siRNA/negative control for 48 h. Interestingly, when the cells were pre-treated with MEKK3 siRNA, no significant difference in p-p65 level was observed regardless of transfection of miR-124 mimic or miR-124 inhibitor was transfected (Fig. 5g). Meanwhile, the RT-qPCR results confirmed that the level of pro-inflammatory cytokines underwent little change regardless of transfection of miR-124 mimic or miR-124 inhibitor (Fig. 5h–j).

### Over-expression of miR-124 or knockdown of MEKK3 could prevent neuronal death and apoptosis following microglial activation in the MCS transfer model

**Fig. 6:**
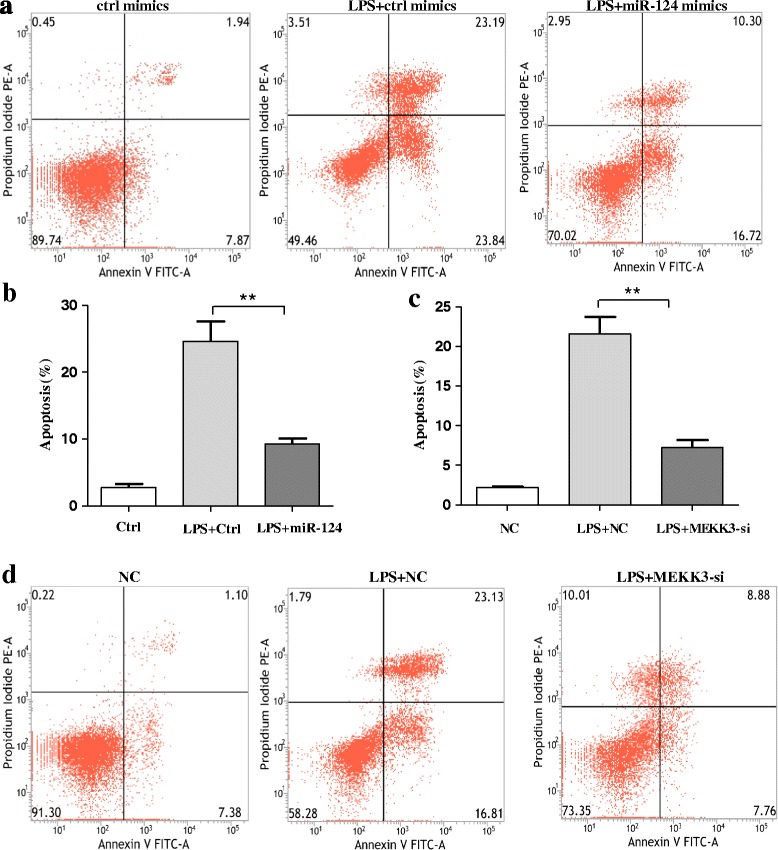
Over-expression of miR-124 or knockdown of MEKK3 could prevent neuronal death and apoptosis following microglial activation in the MCS transfer model. BV2 microglial cells were transfected with miR-124 mimics/control mimics or MEKK3 siRNA/negative control for 48 h. Subsequently, the cells were incubated in the presence of LPS (1 μg/mL) for 12 h. SH-SY5Y cells were cultured normally for 24 h before adding a mixture of BV2-conditioned medium and fresh medium at a ratio of 1:1 (*v*/*v*). After 24 h, the SH-SY5Y cells were harvested. In the miR-124 mimic/control mimic group, the neuronal apoptosis level was assessed using flow cytometry analysis (**a**), and the percentage of apoptotic cells in the total neuronal population was calculated (**b**). In the MEKK3 siRNA/negative control group, the percentage of apoptotic cells in the total neuronal population was calculated (**c**), and the neuronal apoptosis level was assessed using flow cytometry analysis (**d**). Data are normalised to those of saline controls and presented as the mean ± SD of three independent experiments. The fold change is statistically significant. **P < 0.01

In the anti-inflammatory role of over-expression of miR-124 or the knockdown of MEKK3 in activated microglia, as evidenced by our results in BV2 cells, we evaluated the potential modulation of the over-expression of miR-124 or the knockdown of MEKK3 as an anti-inflammatory and neuroprotective strategy. Briefly, BV2 microglial cells were transfected with miR-124 mimics/control mimics or MEKK3 siRNA/negative control for 48 h before exposure to LPS. Subsequently, the cells were incubated in the presence of LPS (1 μg/mL) for another 12 h. Then, the medium of the BV2 cells was collected and mixed with fresh medium at a ratio of 1:1 (*v*/*v*). SH-SY5Y cells were incubated with this conditioned medium for 12 h before the assessment of neurons at the apoptosis level. When miR-124 was over-expressed in BV2 cells, the percentage of apoptotic midbrain DA cells was significantly lower (approximately 15%) than that in the control (Fig. 6a, b). In addition, a similar difference in the apoptotic midbrain DA cell percentage was observed between the negative control (approximately 7%) and the MEKK3 siRNA group (approximately 21.6%), Fig. 6c, d).

### Increasing expression levels of MEKK3, p-p65, and activated microglia are evidenced in the SNpc of MPTP-treated mice in vivo

**Fig. 7:**
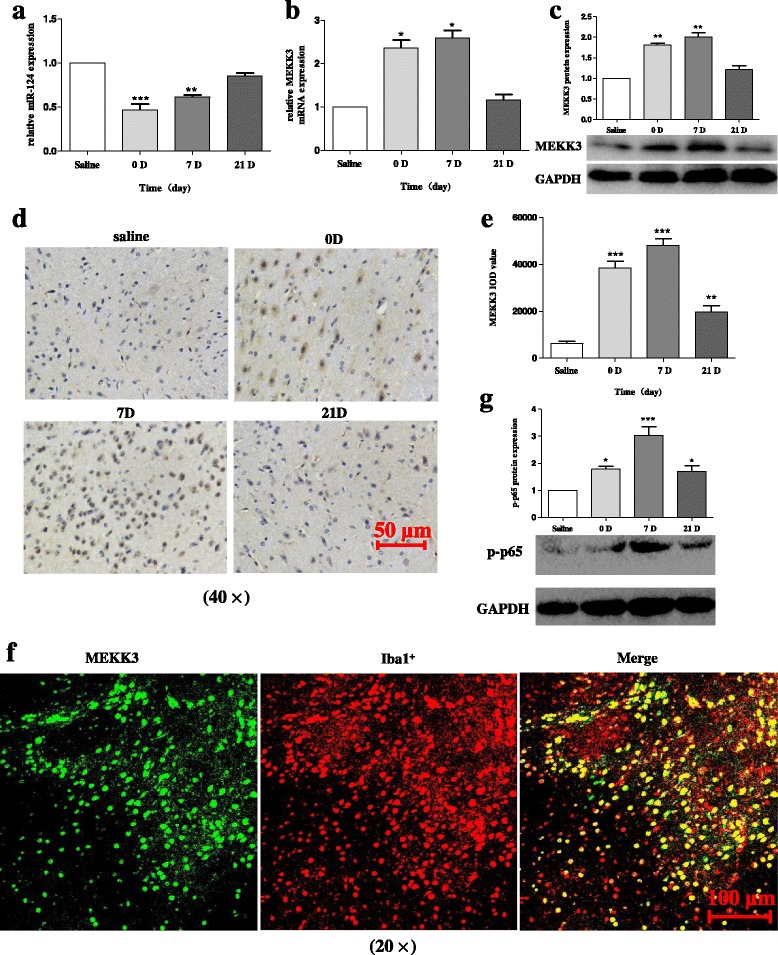
Increasing expression levels of MEKK3, p-p65, and activated microglia are evidenced in the SNpc of MPTP-treated mice in vivo. The mice received one intraperitoneal injection of MPTP-HCl per day for five consecutive days, whereas the control mice received saline injections. Then, the mice were decapitated, and the midbrains were harvested at different time points after MPTP intoxication as follows: 0 (immediately after the last MPTP injection), 1, 7, and 21 days after the last MPTP injection. **a** The miR-124 expression level was determined using RT-qPCR and normalised with U6 RNA. The production mRNA level of MEKK3 (**b**) was determined using RT-qPCR. Western blot analysis evaluated the MEKK3 expression (**c**) in the total protein samples extracted from the midbrain. The data were normalised against GAPDH. **d**, **e** Immunohistochemical analysis was performed to analyse the MEKK3 response (**d**). Graphical representation of the MEKK3 IOD value after MPTP injection is shown (**e**). The scale bar represents 50 μm. **f** A confocal image supported by immunofluorescence confirmed the expression levels of MEKK3 and Iba1^+^. Green: anti-MEKK3; red: anti-Iba1 (antibody microglia). The scale bar represents 100 μm. **g** Western blot analysis determined p-p65 protein expression after MPTP injection. Data are normalised to those of saline controls and presented as the mean ± SD of three independent experiments. The fold change is statistically significant. *P < 0.05, **P < 0.01, ***P < 0.001

We next prepared MPTP-treated mice as a model for PD and further reported on the alteration of the expression levels of miR-124, MEKK3, and p-p65 in the midbrain. In MPTP-treated mice, we first investigated the expression of miR-124 and found that intraperitoneal injection of MPTP could decrease the miR-124 level (Fig. 7a). Subsequently, we observed that the MEKK3 expression at mRNA and protein levels was dramatically upregulated in the MPTP-treated PD model (Fig. 7b, c). Immunohistochemical analysis of the midbrain revealed a significant increase in the MEKK3 expression with MPTP injection (Fig. 7d, e). Further examination of the midbrain by using confocal laser scanning microscopy revealed that MEKK3 was mainly expressed in microglial cells rather than in neuronal cells (Fig. 7f). Besides, the protein level of p-p65 was significantly increased in the MPTP-induced PD model (Fig. 7g). Thus, we believed that MEKK3 is a sign of the neuroinflammation in the PD pathogenic process.

### MPTP injection could induce microglial activation and neuroinflammation in the SNpc of MPTP-treated mice

**Fig. 8:**
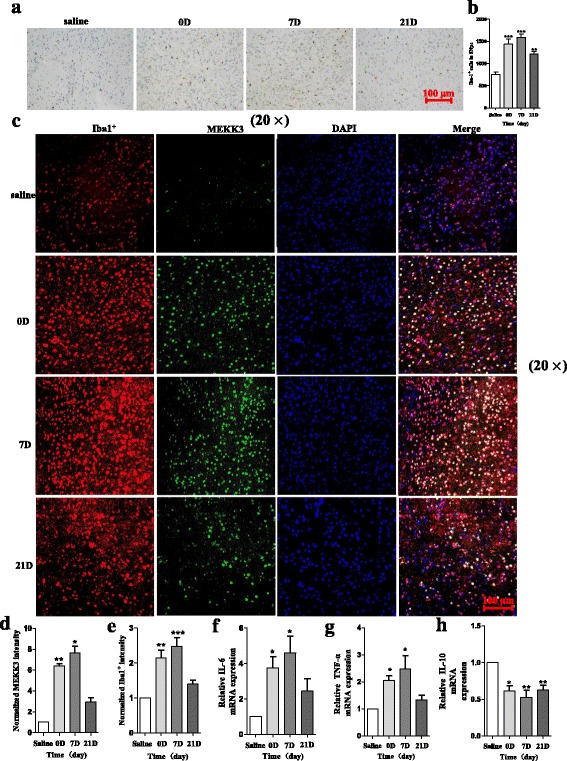
MPTP injection could induce microglial activation and neuroinflammation in the SNpc of MPTP-treated mice. The mice received one intraperitoneal injection of MPTP-HCl per day for five consecutive days, whereas the control mice received saline injections. Then, the mice were decapitated, and the midbrains were obtained at different time points after MPTP intoxication as follows: 0 (immediately after the last MPTP injection), 1, 7, and 21 days after the last MPTP injection. Immunohistochemical analysis was performed to analyse the Iba1 expression (**a**). The total number of Iba1^+^ cells was counted after MPTP injection (**b**). The scale bar represents 100 μm. A representative confocal image showing the expression of MEKK3 and Iba1^+^ is presented (**c**), and graphical representations of MEKK3 (**d**) and Iba1^+^ (**e**) intensity after MPTP injection are also shown. Green: anti-MEKK3; red: anti-Iba1; blue: DAPI. The scale bar represents 100 μm. RT-qPCR confirmed the expression levels of IL-6 (**f**), TNF-α (**g**), and IL-10 (**h**) after MPTP injection, and the fold change was normalised by GAPDH levels. Data are normalised to those in saline controls and presented as the mean ± SD of three independent experiments. The fold change is statistically significant. *P < 0.05, **P < 0.01, ***P < 0.001

Specifically, we examined the microglial activation by immunohistochemical analysis in the PD model and demonstrated that the morphological analysis of Iba1^+^ microglial cells was increased in the SNpc of MPTP-treated mice (Fig. 8a, b). In addition, we observed a parallel increase in the fluorescence intensity of MEKK3 and Iba1 in the MPTP-treated model (Fig. 8c–e). Notably, the mRNA levels of IL-6 and TNF-α were highly increased while the neuroprotective factor IL-10 was decreased in the PD model (Fig. 8f–h). Therefore, MPTP injection could induce microglial activation and neuroinflammation in the SNpc of MPTP-treated mice. It is reasonable to conclude that neuroinflammation induced by activated microglia might play an important role in the PD pathogenic process.

### Exogenous delivery of miR-124 could inhibit the expression of MEKK3 and p-p65 in the SNpc of MPTP-treated mice in vivo

**Fig. 9:**
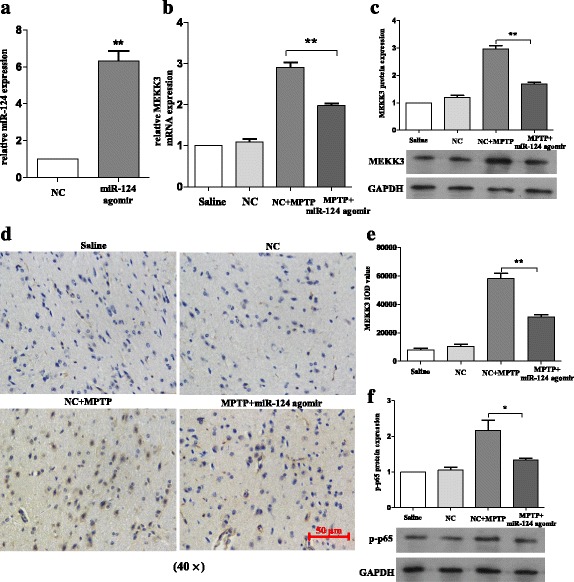
Exogenous delivery of miR-124 could inhibit the expression of MEKK3 and p-p65 in the SNpc of MPTP-treated mice in vivo. The mice were treated with stereotactic intraventricular treatment of miR-124 agomir for five consecutive days. Next, the mice received one intraperitoneal injection of MPTP-HCl per day for 5 days, while the control mice received saline injections. Agomir treatment was performed 2 days prior to the injection of MPTP. Then, the mice were decapitated, and the midbrains were obtained 7 days after the last MPTP injection. **a** The miR-124 expression was evaluated using RT-qPCR and normalised with U6 RNA. In the agomir-treated mice and their negative control counterparts on day 7 after the last injection of MPTP, RT-qPCR was used to determine the production mRNA levels of MEKK3 (**b**), and western blot analysis was used to evaluate the MEKK3 (**c**) expression in total protein samples extracted from the midbrain. The fold change was normalised by GAPDH levels. Immunohistochemical analysis was performed to analyse the MEKK3 response (**d**), and a graphical representation of MEKK3 IOD value (**e**) from the midbrain is shown. The scale bar represents 50 μm. **f** Western blot analysis was used to determine the p-p65 protein expression. Data are normalised to saline controls and presented as the mean ± SD of three independent experiments. The fold change is statistically significant. *P < 0.05, **P < 0.01

Intrigued by the fact that miR-124 attenuates LPS-induced inflammatory responses by MEKK3/NF-κB signalling pathways in vitro, we investigated whether miR-124 could mediate the activation of microglia in MPTP-treated mice in vivo. Accordingly, considering the neuroprotective effect of miR-124 in other CNS diseases [19], miR-124 agomir or negative control was injected into the right lateral ventricle 2 days before MPTP treatment to upregulate the miR-124 expression in the midbrain. Then, the exogenous delivery efficacy of miR-124 agomir was assessed using RT-qPCR (Fig. 9a). It is interesting that we observed that upregulation of miR-124 could inhibit the expression of MEKK3 and p-p65 in vivo, which was consistent with in vitro results (Fig. 9b–f). Consequently, exogenous delivery of miR-124 could inhibit the expression levels of MEKK3 and p-p65 in the SNpc of MPTP-treated mice.

### Exogenous delivery of miR-124 attenuates the activation of microglia in the SNpc of MPTP-treated mice in vivo

**Fig. 10:**
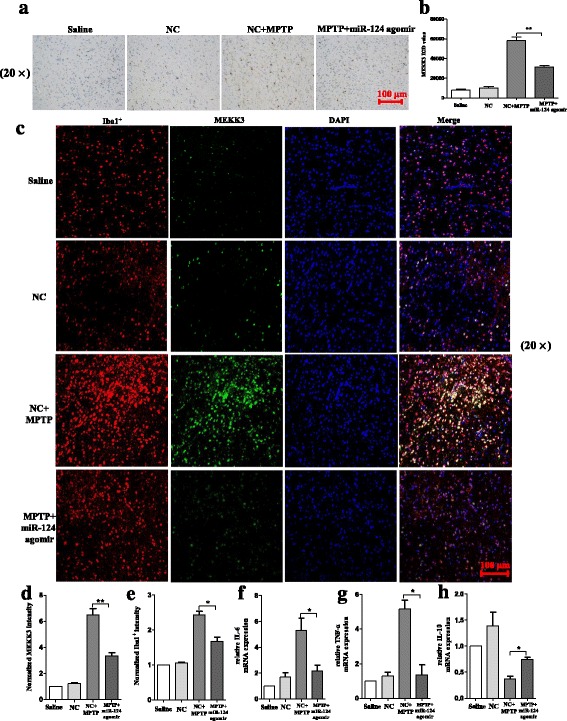
Exogenous delivery of miR-124 attenuates the activation of microglia in the SNpc of MPTP-treated mice in vivo. The mice were treated with stereotactic intraventricular treatment of miR-124 agomir for five consecutive days. Next, the mice received one intraperitoneal injection of MPTP-HCl per day for 5 days, whereas the control mice received saline injections. The agomir treatment was performed 2 days prior to the MPTP injection. Then, the mice were decapitated, and the midbrain was obtained 7 days after the last MPTP injection. Immunostaining (**a**) and stereological counts (**b**) of Iba1^+^ cells in the SNpc are shown. The scale bar represents 100 μm. A confocal image of MEKK3 and Iba1 is shown (**c**), and a graphical representation of MEKK3 (**d**) and Iba1 (**e**) intensity in the SNpc is presented. Green: anti-MEKK3; red: anti-Iba1; blue: DAPI. The scale bar represents 100 μm. **f**–**h** Western blot analysis determined the p-p65 protein expression, and RT-qPCR confirmed the expression levels of IL-6 (**f**), TNF-α (**g**), and IL-10 (**h**) from the midbrain. The fold change was normalised by GAPDH levels. Data are normalised to saline controls and presented as the mean ± SD of three independent experiments. The fold change is statistically significant. *P < 0.05, **P < 0.01

Correspondingly, the injection of miR-124 agomir in the MPTP-treated mice attenuated the activation of microglia in the SNpc in comparison with the negative control (Fig. 10a, b). In addition, compared with the negative group, a parallel decrease was observed in the fluorescence intensity of MEKK3 and Iba1^+^ in the miR-124 agomir group (Fig. 10c–e). Besides, the mRNA levels of the pro-inflammatory cytokines IL-6 and TNF-α were highly decreased, while that of the neuroprotective factor IL-10 increased considerably with injection of miR-124 agomir (Fig. 10f–h). These data indicate that exogenous delivery of miR-124 could suppress the activation of microglia in the SNpc of MPTP-treated mice in vivo.

### miR-124 attenuates MPTP-dependent apoptotic midbrain DA cell death in vivo

**Fig. 11:**
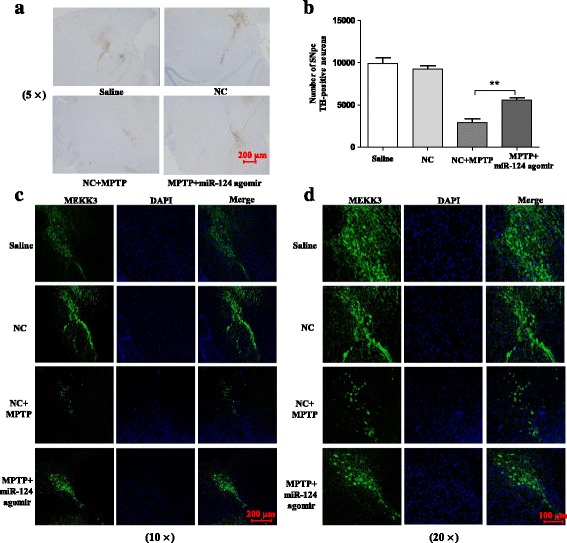
miR-124 attenuates MPTP-dependent apoptotic midbrain DA cell death in vivo. The mice were treated with stereotactic intraventricular treatment of miR-124 agomir for five consecutive days. Next, the mice received one intraperitoneal injection of MPTP-HCl per day for 5 days, whereas the control mice received saline injections. The agomir treatment was performed 2 days prior to the MPTP injection. Then, the mice were decapitated, and the midbrain was obtained 7 days after the last MPTP injection. Immunostaining (**a**) and stereological counts (**b**) of TH-positive neurons in the SNpc are shown. The scale bar represents 200 μm. Low magnification (scale bar, 200 nm) (**c**) and high magnification (scale bar, 100 nm) (**d**) are supported by confocal laser scanning microscopy of TH-positive neurons. Data are presented as the mean ± SD of three independent experiments. The fold change is statistically significant. **P < 0.01

Having found that miR-124 could inhibit neuroinflammation in the animal model of PD, we investigated whether over-expression of miR-124 could prevent midbrain DA neuronal death in vivo. Interestingly, we finally observed that over-expression of miR-124 in the midbrain could prevent midbrain DA neuronal death and apoptosis in the SNpc of MPTP-treated mice (Fig. 11a–d). These results demonstrated that exogenous delivery of miR-124 offers strong neuroprotection in the PD pathogenic process.

## Discussion

Increasing evidence has demonstrated that miRNAs play pivotal roles in neuron biology and that miR-124 is highly expressed in the brain and during CNS development [39, 40]. Studies have demonstrated that miR-124 may alleviate neuron death in diverse types of neurodegeneration diseases, including Alzheimer’s disease, Parkinson’s disease, amyotrophic lateral sclerosis, and multiple sclerosis [16, 41]. In addition, miR-124 expression is downregulated in neurons from the MPTP-induced PD model [42]. Evidence shows that miR-124 is a critical mediator for the peripheral and CNS inflammatory process by inhibiting the activation of microglia/macrophages and reducing the production of pro-inflammatory cytokine [20, 43]. However, the underlying mechanism is still unclear. Notably, we would demonstrate the potential neuroprotective role of miR-124 in both cell lines and a pre-clinical mouse model for PD. In our study, we found that miR-124 was downregulated in the LPS-stimulated BV2 cells in a dose- and time-dependent manner, which is consistent with previous reports that miR-124 is a key regulator of microglial cell quiescence in the CNS [19]. We then suggested that decreasing miR-124 levels may be necessary for the progression of microglial cell activation and the production of inflammatory mediators. An increased level of miR-124 following transfection with miR-124 mimics in BV2 cells resulted in a significant reduction in the expression levels of the neurotoxic cytokines iNOS, IL-6, and TNF-α and in a high increase in the levels of the anti-inflammatory cytokines TGF-β1 and IL-10. Meanwhile, the expression levels of IL-6 and TNF-α were highly increased while the neuroprotective factor IL-10 was decreased after intraperitoneal injection of MPTP. Specifically, our data for exogenous delivery of miR-124 showed the same trend in MPTP-induced inflammatory response in vivo, which was consistent with in vitro experiments. However, we obtained the opposite result when transfecting with the miR-124 inhibitor in comparison with the miR-124 mimics. Consequently, these data indicate that the over-expression of miR-124 could effectively attenuate LPS- or MPTP-induced microglial activation in vitro or in vivo.

Accordingly, MEKK3, an inflammation-associated protein that has been identified as a target of miR-124 in our study, is upregulated in the MPTP-induced PD model. However, the same trend is found for the expression of MEKK3 in activated microglial cells that were stimulated by the Toll-like receptor 4 ligand LPS. Specifically, we then found that MEKK3 was mainly expressed in microglial cells rather than in neuronal cells and that its expression is significantly elevated with microglial activation in vivo. To further clarify the role of MEKK3 in the microglia inflammatory processes, we transfected MEKK3 siRNA into the BV2 cells. The results reflected that the expression levels of TNF-α, iNOS, and IL-6 were significantly decreased. Moreover, the knockdown of MEKK3 suppressed the activity of NF-κB in microglia, which agrees with previous studies that MEKK3 can mediate NF-κB activation [21]. Nevertheless, we observed that the transfection of miR-124 mimic to microglial cells could counter-regulate the MEKK3 upregulation induced by LPS in vitro, as miR-124 targeting MEKK3 was demonstrated by the luciferase reporter assay in our study. We further treated mice with MPTP and found that an increasing expression level of MEKK3 was evidenced in the SNpc, whereas we observed a similar result in a silencing MEKK3 gene study where MEKK3 expression level was also reduced by upregulation of miR-124 in vivo. Therefore, our data identified MEKK3 as a target of miR-124. Correspondingly, we observed a parallel alteration of MEKK3 and Iba1^+^ in both MPTP-induced and agomir-treated mice, and we considered that MEKK3 was a sign of the neuroinflammation.

The NF-κB, which is a ubiquitous transcription factor that regulates immune and cell survival signalling pathways, plays a pivotal role in both inflammatory response and cell survival [44]. NF-κB consists of a group of seven transcription factors and is known for its crucial transcription factors that regulate the expression levels of pro-inflammatory cytokines such as TNF-α, iNOS, and IL-6 [45, 46]. Currently, the activation of NF-κB has been reported in the CNS of neurodegenerative diseases in animal models and human patients [47]. Several studies support the hypothesis that the activation of NF-κB p65 plays an important role in PD pathogenesis and that the selective inhibition of NF-κB protected dopaminergic neurons from MPTP toxicity [46]. NF-κB has been found to be activated in the SNpc in a hemiparkinsonian monkey model of PD and that the inhibition of NF-κB could supress the secretion of pro-inflammatory molecules, protect the midbrain DA cells from death, and improve locomotor activity [48]. In fact, treatment with LPS can significantly increase the NF-κB activity in microglial cells, and morphine alters the LPS-induced activation of NF-κB through PKCε-Akt-ERK1/2 signalling [49]. In our study, we determined that the expression of p-p65 was obviously increased in LPS-stimulated microglial cells and in MPTP-induced PD model. In addition, several miRNAs have been reported to modify cell behaviour by regulating the NF-κB pathway. MicroRNA-26b suppresses NF-κB signalling by targeting TAK1 and TAB3, which is a potent inhibitor of the NF-κB pathway [50]. The miR-146 levels were downregulated, and the upregulation of miR-146 expression may be of neuroprotective value in AD, whereas the levels of its target proteins IL-1 receptor-associated kinase-1 and NF-κB increased in the microglial cells of PS-2 knockout mice [51]. Clearly, the identification of miRNAs that target NF-κB signalling may provide novel molecular targets for disease therapy. On the basis of the above-mentioned studies, we examined the anti-inflammatory properties of miR-124 and further studied the potential mechanisms of its neuroprotective effect. Our results showed that over-expression of miR-124 significantly suppressed the expression of LPS- or MPTP-induced upregulation and the activation of p-p65 in protein level. In particular, we considered that miR-124 could mediate the NF-κB signalling pathway by regulating the expression of MEKK3, which might be a therapeutic target in inflammatory responses. Meanwhile, we found no significant difference in the p-p65 levels of the pro-inflammatory cytokines regardless of transfection of miR-124 mimic or miR-124 inhibitor when the cells were pre-treated with MEKK3 siRNA, demonstrating that miR-124 regulates the LPS-induced secretion of pro-inflammatory cytokines and NF-κB in microglial cells, at least partly, through the regulation of MEKK3.

Finally, we found that the apoptosis and death of SH-SY5Y cells could be suppressed when the microglial activation was inhibited by the upregulation of miR-124 or the knockdown of MEKK3 in MCS. Meanwhile, the exogenous delivery of miR-124 could attenuate the activation of microglia in MPTP-treated mice and prevent MPTP-dependent apoptotic midbrain DA cell death in vivo. The observed increase in the neuronal apoptosis rate most likely results from the decreased levels of the inflammatory cytokines present in the conditioned medium because direct treatment of the neuron with LPS did not affect cell apoptosis. Thus, it was reasonable that neuroinflammation induced by activated microglia might play an important role in the PD pathogenic process.

## Conclusion

In conclusion, we found that miR-124 expression was downregulated in LPS-treated BV2 cells and could suppress the secretion of pro-inflammatory mediators by targeting the MEKK3/NF-κB signalling pathways. Moreover, MEKK3 and p-p65 were abundantly expressed, and microglia were activated in the SNpc in the MPTP model of PD. Furthermore, exogenous delivery of miR-124 could suppress MEKK3 and p-p65 expression levels and attenuate the activation of microglia in MPTP-treated mice. Taken together, our data suggest that miR-124 can inhibit neuroinflammation that occurs in the development of PD and implicate miR-124 as a potential therapeutic target for regulating the inflammatory response in PD.

## Abbreviations

3′-UTR: 3′ untranslated regions
CNS: Central nervous system
DA: Dopaminergic
GAPDH: Glyceraldehyde-3-phosphate dehydrogenase
HtrA2: HtrA serine peptidase 2
IL-10: Interleukin-10
IL-6: Interleukin-6
iNOS: Inducible nitric oxide synthase
IOD: Integrated optical density
LPS: Lipopolysaccharide
MCS: Microglial culture supernatant
MEKK3/MAP3K: Mitogen-activated protein kinase kinase kinase
MEKK3-si: MEKK3 small interfering RNA
miR-124: MicroRNA-124
miRNAs: MicroRNAs
MPTP: 1-Methyl-4-phenyl-1,2,3,6-tetrahydropyridine
NC: Negative control
NF-κB: Nuclear factor of kappaB
p65: RelA
PD: Parkinson’s disease
p-p65: Phosphorylation of NF-κB p65
RIPA: Radioimmunoprecipitation assay
ROS: Reactive oxygen species
RT-qPCR: Reverse transcription quantitative real-time polymerase chain reaction
TGF-β1: Transforming growth factor beta 1
TH: Tyrosine hydroxylase
TNF-α: Tumour necrosis factor alpha
